# Panels of tumor-derived RNA markers in peripheral blood of patients with non-small cell lung cancer: their dependence on age, gender and clinical stages

**DOI:** 10.18632/oncotarget.10558

**Published:** 2016-07-13

**Authors:** Chih-Feng Chian, Yi-Ting Hwang, Harn-Jing Terng, Shih-Chun Lee, Tsui-Yi Chao, Hung Chang, Ching-Liang Ho, Yi-Ying Wu, Wann-Cherng Perng

**Affiliations:** ^1^ Division of Pulmonary and Critical Care Medicine, Department of Internal Medicine, Tri-Service General Hospital, National Defense Medical Center, Taipei, Taiwan, ROC; ^2^ Department of Statistics, National Taipei University, Taipei, Taiwan, ROC; ^3^ Advpharma, Inc., Taipei, Taiwan, ROC; ^4^ Division of Thoracic Surgery, Department of Surgery, Tri-Service General Hospital, National Defense Medical Center, Taipei, Taiwan, ROC; ^5^ Division of Hematology and Oncology, Department of Internal Medicine, Taipei Medical University-Shuang Ho Hospital, Taipei, Taiwan, ROC; ^6^ Division of Hematology and Oncology, Department of Internal Medicine, Tri-Service General Hospital, National Defense Medical Center, Taipei, Taiwan, ROC; ^7^ Graduate Institute of Life Sciences, National Defense Medical Center, Taipei, Taiwan, ROC

**Keywords:** circulating tumor cells, gene expression profiling, non-small cell lung cancer

## Abstract

Peripheral blood mononuclear cell (PBMC)-derived gene signatures were investigated for their potential use in the early detection of non-small cell lung cancer (NSCLC). In our study, 187 patients with NSCLC and 310 age- and gender-matched controls, and an independent set containing 29 patients for validation were included. Eight significant NSCLC-associated genes were identified, including *DUSP6*, *EIF2S3*, *GRB2*, *MDM2*, *NF1*, *POLDIP2*, *RNF4*, and *WEE1*. The logistic model containing these significant markers was able to distinguish subjects with NSCLC from controls with an excellent performance, 80.7% sensitivity, 90.6% specificity, and an area under the receiver operating characteristic curve (AUC) of 0.924. Repeated random sub-sampling for 100 times was used to validate the performance of classification training models with an average AUC of 0.92. Additional cross-validation using the independent set resulted in the sensitivity 75.86%. Furthermore, six age/gender-dependent genes: *CPEB4*, *EIF2S3*, *GRB2*, *MCM4*, *RNF4*, and *STAT2* were identified using age and gender stratification approach. *STAT2* and *WEE1* were explored as stage-dependent using stage-stratified subpopulation. We conclude that these logistic models using different signatures for total and stratified samples are potential complementary tools for assessing the risk of NSCLC.

## INTRODUCTION

Lung cancer is one of the leading causes of cancer mortality worldwide, with an overall 5-year survival rate of only 13% in Europe [1] and 17% in the US [2]. Although the lung cancer death rate is decreasing in the US, it has been increasing in some Asian countries such as China [3]. In 2015, an estimated 221,200 new cases and 158,040 deaths of lung cancer are expected in the US. Symptoms do not usually occur until the cancer is in the advanced stages, and more than 60% and 80% of patients with lung cancer were found to have locally advanced or distant metastasis at initial diagnosis in the US and Taiwan, respectively [4, 5]. Although new-generation chemotherapeutic agents and targeted therapies have been introduced, the 5-year overall survival rates are still unsatisfactory, at 9–24% for stage IIIB and 1–4% for stage IV [6]. These rates highlight the importance of early detection to improve the overall survival for patients with lung cancer. Annual screening by low-dose chest computed tomography (LDCT) is currently undergoing a lot of flux and has shown a 20% reduction in the mortality rate [7]. However, the specificity of LDCT for the detection of lung cancer was 73.4% in screening centers with experienced staffs [8]. The application of lung cancer biomarkers could provide an easier and routine method for early detection of lung cancer. Previous studies on cancer biomarkers have shifted from the analysis of mutations [9], gene copy number variations [10], expression alterations and epigenetic regulation [11] to the analysis of gene or protein signatures.

Gene expression profiling of lung tumors and their adjacent normal tissues for early detection, monitoring and prognosis has resulted in a new perspective in recent decades [12]. Particularly, nucleic acid-based biomarkers and their changes in the peripheral blood have been studied for their usefulness in the early detection of lung cancer. A few previous studies have focused on the expression of single or multiple genes in peripheral blood; however, most of these studies resulted in unsatisfactory diagnostic performance [13–15]. Single marker-based assays generally have low sensitivities (30%–64%) [13–16]. The use of multiple markers could improve test sensitivity by up to 72%–85% [16, 17], and have become popular in the development of cancer diagnostic and prognostic assays [17, 18]. In addition, lung cancer incidence increases markedly after the age of 45–54 years, peaking among those aged >75 years for both sexes [19]; however, samples from healthy controls in published reports are generally limited in terms of information on age, gender, and smoking history as well as other demographic factors.

We focused on the discovery of specific nucleic acid-based markers in peripheral blood mononuclear cell (PBMC) fractions from patients with non-small cell lung cancer (NSCLC). The clinical utilities of the expression of the investigated genes were evaluated in newly diagnosed patients with NSCLC and age- and gender-matched non-cancer controls. Several multiple logistic models for total sample, age and gender-, and clinical stage-stratified subpopulations were conducted to explore demographic- and stage-dependent markers.

## RESULTS

The present case–control study was conducted on blood samples from 187 patients with NSCLC and 310 gender- and age-matched non-cancer controls. Among all the participants, 58.27% were male, 57.48% were ≥66 years of age and 39.17% were smokers. There were no statistically significant differences in gender or age between the NSCLC cases and non-cancer controls. However, participants who smoked were more likely to be in the case group (55.08%) than in the control group (29.68%) (*p* < 0.0001) (Table 1).

**Table 1:** Characteristics of the study sample (*N* = 497)

	NSCLC Cases		Non-cancer Control		Total Sample		*p*^§^
	*n*	%	*n*	%	*n*	%	
Sample size	187	37.63	310	62.37	497	100.00	
Gender							
Female	73	39.04	134	43.23	212	41.73	0.3588
Male	114	60.96	176	56.77	296	58.27	
Age							
36-65	81	43.32	127	40.97	216	42.52	0.6073
66-95	106	56.68	183	59.03	292	57.48	
Smoking status*							
No	84	44.92	218	70.32	309	60.83	<0.0001
Yes	103	55.08	92	29.68	199	39.17	

§ The *p* value was obtained from the chi-square test.

* Smoking status: No: non-smoker, Yes: current smoker and ever smoker.

### Identification of differentially expressed genes between NSCLC cases and controls

The bivariate associations between the mean relative expression of 15 investigated genes and cancer are shown in Table 2. Statistically significant differences were found in 11 of the 15 genes between NSCLC cases and non-cancer controls in this study. In particular, the mean relative expression levels of the *EIF2S3*, *EXT2*, *RNF4*, and *WEE1* genes for NSCLC cases were significantly lower than those for non-cancer controls, whereas the mean relative expression levels of seven genes, namely *CPEB4*, *DUSP6*, *GRB2*, *MCM4*, *MDM2*, *MMD*, and *STAT2*, were significantly higher for NSCLC cases than for non-cancer controls.

**Table 2:** Analysis of bivariate association of the relative mean expression of 15 investigated genes between NSCLC cases and non-cancer controls

Gene	Total Sample		NSCLC Cases		Non-cancer Controls		*p*^§^
	Mean	SD	Mean	SD	Mean	SD	
*Significant Lower Mean Expression in NSCLC Cases*							
*EIF2S3*	3.56	0.53	3.46	0.65	3.61	0.43	0.0028***
*EXT2*	−0.71	0.65	−0.83	0.74	−0.63	0.58	0.0008***
*RNF4*	2.03	0.58	1.92	0.58	2.09	0.57	0.0017***
*WEE1*	−0.59	0.67	−0.86	0.71	−0.42	0.59	0.0000***
*Significant Higher Mean Expression in NSCLC Cases*							
*CPEB4*	0.75	1.03	1.14	1.13	0.51	0.88	0.0000***
*DUSP6*	1.93	0.81	2.47	0.79	1.61	0.63	0.0000***
*GRB2*	2.16	0.79	2.44	0.84	1.99	0.71	0.0000***
*MCM4*	−1.84	0.68	−1.73	0.72	−1.90	0.65	0.0093***
*MDM2*	−0.76	0.64	−0.49	0.72	−0.92	0.52	0.0000***
*MMD*	1.61	1.06	1.79	1.22	1.50	0.93	0.0034***
*STAT2*	1.88	0.59	1.99	0.68	1.81	0.52	0.0008***
*No Significant Difference of Mean Expression Between Two Groups*							
*IRF4*	0.23	0.69	0.23	0.72	0.23	0.67	0.9488
*NF1*	0.86	0.47	0.82	0.57	0.88	0.40	0.2078
*POLDIP2*	2.42	0.70	2.46	0.74	2.40	0.67	0.4122
*ZNF264*	−2.33	0.58	−2.29	0.62	−2.36	0.54	0.1998

§ The *p* value was obtained from the independent two-sample *t*-test.

***: The gene expression level between NSCLC cases and non-cancer controls was significantly different (*p* < 0.05).

### NSCLC-associated molecular markers in PBMC-derived fractions and classification model

Logistic regression analysis was applied to construct a lung cancer molecular (LCM) model containing all 15 of the investigated genes with controlling for age, gender and smoking history to assess the participants' risk for developing lung cancer. In this model, the relative expression levels of eight genes were significantly associated with lung cancer after controlling for basic demographics (Table 3). Interestingly, the *NF1* and *POLDIP2* genes were found to be significant factors in the logistic model but not in the marginal analysis based on the independent two-sample *t*-test.

**Table 3:** Multivariate analysis and selection of significant NSCLC-associated molecular markers in the total sample (N = 497). #*

Gene	OR	95%CI of OR		*p*	StdEst
*DUSP6*	7.71	4.20	14.13	0.0000	0.91
*GRB2*	7.41	3.63	15.13	0.0000	0.87
*MDM2*	5.36	2.47	11.65	0.0000	0.59
*EIF2S3*	0.22	0.10	0.48	0.0002	−0.45
*NF1*	0.35	0.14	0.88	0.0255	−0.27
*POLDIP2*	0.16	0.07	0.35	0.0000	−0.71
*RNF4*	0.22	0.09	0.55	0.0014	−0.49
*WEE1*	0.47	0.25	0.86	0.0150	−0.28
C statistic	0.924				

OR: odds ratio; CI: confidence interval; StdEst: standardized coefficients;

# The multiple logistic model contains 15 expressed genes, with controlling for age, gender and smoking status. The other seven molecular markers (*CPEB4*, *EXT2*, *IRF4*, *MCM4*, *MMD*, *STAT2* and *ZNF264*) were not significantly associated with NSCLC.

* The performance of this model is presented as the sensitivity and specificity depending on the cut-off value chosen, for instance:

Cut-off value = 0.434, sensitivity = 0.807, specificity = 0.906

Cut-off value = 0.321, sensitivity = 0.861, specificity = 0.855

Cut-off value = 0.226, sensitivity = 0.904, specificity = 0.774

According to the LCM model, participants who had relative higher expression of the *DUSP6*, *GRB2*, and *MDM2* genes and relative lower expression levels of the *EIF2S3*, *NF1*, *POLDIP2*, *RNF4*, and *WEE1* genes were more likely to be in the case group. For each unit increase in the relative expression of the *DUSP6*, *GRB2*, and *MDM2* genes, the odds of having lung cancer increased by 7.71, 7.41 and 5.36, respectively. Each unit increase in the relative expression of the *EIF2S3*, *POLDIP2*, and *RNF4* genes showed protective effects, with odds of having lung cancer decreased by 78%, 84%, and 78%, respectively. In addition, each unit increase in the relative expressions of the *NF1* and *WEE1* genes presented slightly weaker protection, with the odds decreased by 65% and 53%, respectively. Overall, expression of *DUSP6* gene had the strongest effect on the prediction of lung cancer based on the absolute value of the standardized coefficients (StdEst).

**Figure 1:**
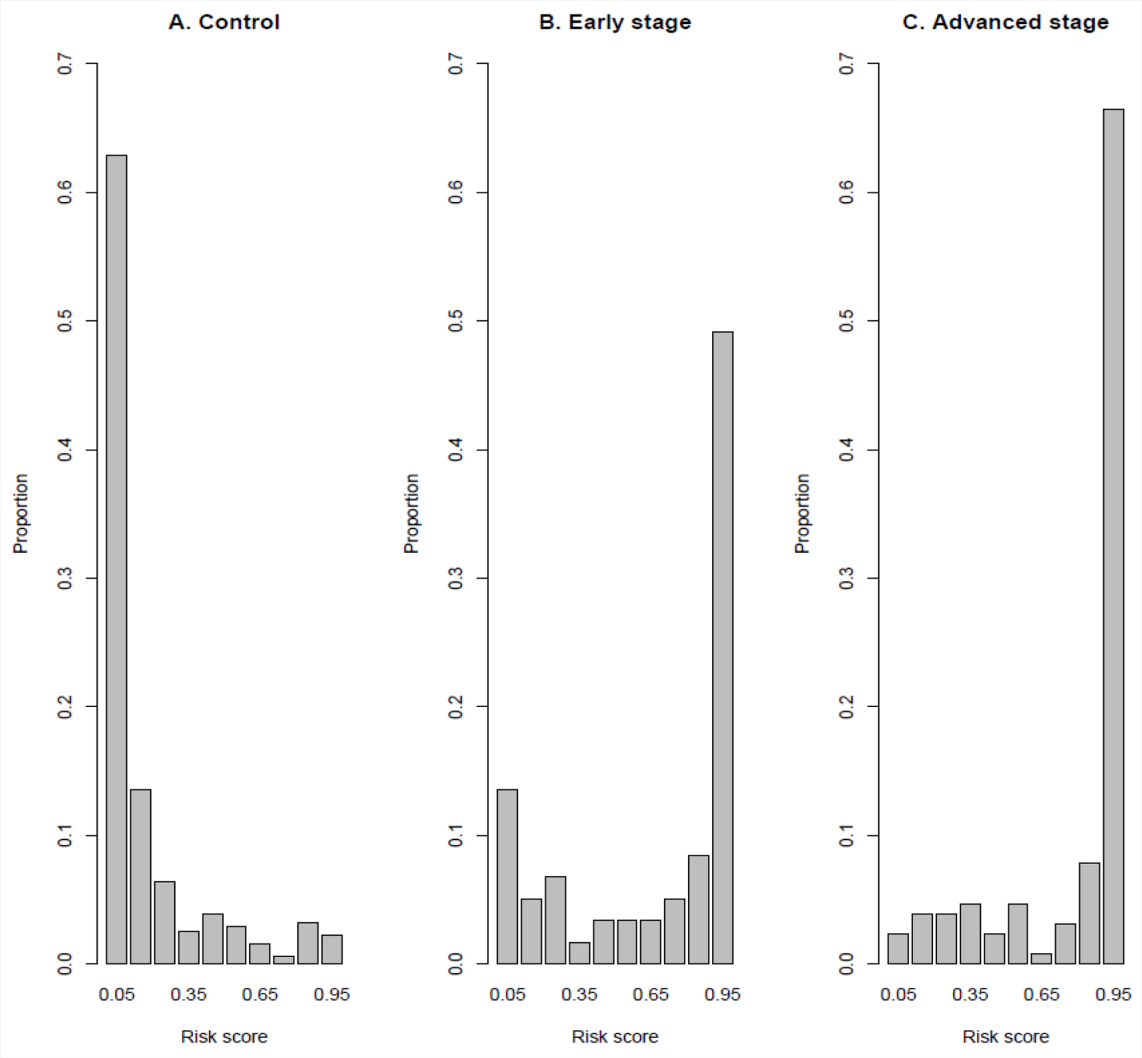
Histogram of risk score of samples (Proportion) **A.** Controls; **B.** Cases with early stage disease and **C.** Cases with advanced stage disease. The risk score is calculated using LCM classification model (Table 3).

The *C* statistic was excellent for the LCM model for classification of patients with NSCLC in all clinical stages and non-cancer controls (area under the curve, AUC = 0.924; Supplementary Figure S1). Particularly, the model yielded 80.7% sensitivity and 90.6% specificity if a cutoff (risk score; probability of developing NSCLC) value of 0.434 was chosen (Table 3). A histogram of risk scores by samples clearly showed the very good performance of classification (Figure 1). The sensitivity was 83.6% and 69.5% for patients with advanced stage (IIIB-IV) and for patients with early stage (I-IIIA), respectively, if a risk score of 0.434 was chosen as cutoff. As expected, most of control subjects (76.5%) had very low risk score ranged 0-0.2.

### Cross-validation of classification model

We applied repeated random sub-sampling method to evaluate how well the classification model generalized and verify the performance of our results. Among 15 genes, six genes (*DUSP6*, *EIF2S3*, *GRB2*, *MDM2*, *POLDIP2*, and *RNF4*) were identified as the most significant factors (*p* values < 0.001) for all training models constructed from 100 random samples. In addition, *NF1* and *WEE1* were also identified as significant factors in 86% and 69% of 100 training models, respectively. These results demonstrated that these eight markers showing significant association with NSCLC were consistent with the LCM model (Table 3) using the total sample. Moreover, each training model was tested using testing data (*N* = 50) for each random sub-sampling. There were a total of 5000 testing data after 100 times sub-sampling. The average AUC from 100 samples was excellent (0.92), as was the classification model using the total sample. In addition, the performances of training models were evaluated with five cutoff values, including 0.622, 0.5, 0.434, 0.321, and 0.226 (Supplementary Table S1). The average sensitivity ranged from 73.1% to 86.5%, and the average specificity was between 90.8% and 76.6%, corresponding to these five cutoff values. The predictive accuracy was over 80% (81.9–84.1%). In summary, the overall performance of training models for cross-validation was very good.

An independent sample was additionally included for validation. This validation set contained 29 cases with early stage (I-II) of NSCLC disease. There were 19 (65.52%) female, 14 (48.28%) cases younger than 65 years old, and 10 (34.48%) smokers. The sensitivity for this independent validation set was 75.86% based on the classification model for total sample (Table 3), when the cutoff value set at 0.434.

### Age- and gender-dependent NSCLC-associated molecular markers

We further stratified participants into four subpopulations to explore age- and gender-dependent molecular markers of lung cancer. Groups based on different genders (men and women) and ages with the cut-off at 65 years were analyzed separately. Four logistic models after controlling for smoking history resulted in four different gene signatures on the basis of 11 NSCLC-associated markers (Table 4). Overall, three significant markers, the *CPEB4*, *MCM4*, and *STAT2* genes, were selected in addition to those for the LCM model. The expression of *DUSP6* gene was the only common factor showing significant associations with NSCLC in all four subpopulation models. The other 10 significant markers included three age-dependent markers (*MDM2*, *NF1*, and *WEE1*), one male-dependent marker (*POLDIP2*), and six age/gender-dependent markers (*CPEB4*, *EIF2S3*, *GRB2*, *MCM4*, *RNF4*, and *STAT2*) (Table 4). The *C* statistic showed excellent performance (AUC > 0.9) for the three age/gender-dependent models based on female and old men subpopulations. The model for the younger men subpopulation was almost excellent (AUC = 0.895).

**Table 4:** Multivariate analysis and selection of NSCLC-associated molecular markers based on age- and gender-stratified subpopulations. #

	Younger women N=100				Older women N=107				Younger men N=108				Older men N=182			
N(Case)	39				34				42				72			
N(Control)	61				73				66				110			
Gene	OR	95%	CI	StdEst	OR	95%	CI	StdEst	OR	95%	CI	StdEst	OR	95%	CI	StdEst
*CPEB4*													5.00	2.09	11.97	0.97
*DUSP6*	4.25	1.69	10.65	0.66	13.64	4.23	44.06	1.29	4.13	1.74	9.79	0.63	34.21	6.67	175.39	1.46
*GRB2*													20.33	4.99	82.77	1.37
*MCM4*					6.31	1.83	21.84	0.70								
*MDM2*	4.81	1.30	17.82	0.51					7.34	1.99	27.05	0.73				
*EIF2S3*									0.21	0.05	0.97	−0.45				
*NF1*					0.10	0.02	0.47	−0.55					0.10	0.02	0.46	−0.63
*POLDIP2*									0.28	0.09	0.86	−0.49	0.01	0.00	0.08	−1.77
*RNF4*					0.16	0.04	0.64	−0.54								
*STAT2*	0.26	0.07	0.94	−0.39												
*WEE1*	0.07	0.02	0.27	−0.96					0.26	0.09	0.76	−0.51				
C statistic	0.907				0.930				0.895				0.970			

OR: odds ratio; CI: confidence interval; StdEst: standardized coefficients

# Each multiple logistic model contains significant genes with controlling for smoking status.

The influence of the expression of *DUSP6* gene, was stronger in the older populations in terms of the odds of having lung cancer. The odds ratio increased from 4.25 to 13.64 and from 4.13 to 34.21 for women and men, respectively, for every unit increase in the relative expression of the *DUSP6* gene. Moreover, the *DUSP6* gene was the most important factor in the model for the older women (StdEst = 1.29).

Among the three age-dependent markers, an increased relative expression of *NF1* and *WEE1* genes had a protective effect on subjects, whereas an increased relative expression of *MDM2* gene was a risk factor for developing lung cancer. In particular, the expression of *NF1* gene presented as a significant and strong protective predictor only for the elderly in our analysis. For every unit increase in the relative expression of *NF1* gene, the odds of having lung cancer dropped by approximately 90% (OR = 0.1). Relative expression levels of the *WEE1* and *MDM2* genes were common significant predictors for the younger groups but had different effects depending on the gender. An increased relative expression of *WEE1* gene decreased the odds of having lung cancer by 74% for younger men and by 93% for younger women. In addition, an increased relative expression of *MDM2* gene was a stronger risk factor for younger men (OR = 7.34) than for younger women (OR = 4.81). The expression of *WEE1* gene (StdEst = −0.96) was the most important predictor in the model for the younger women, whereas the expression of *MDM2* gene (StdEst = 0.73) was the most important predictor in the model for the younger men.

The only significant gender-dependent predictor was the expression of *POLDIP2* gene in men and its effect was stronger in the older group. For each unit increase in the relative expression of *POLDIP2* gene, the odds of having lung cancer decreased by 72% and 99% for younger and older men, respectively. Furthermore, the *POLDIP2* gene was the most influential predictor in the model for older men (StdEst = −1.77).

The other six significant age/gender-dependent NSCLC-associated markers found had particular specificities for each stratified sample. In terms of logistic models for the female subpopulations, the expression of *STAT2* gene for younger women was significant, whereas the *MCM4* and *RNF4* genes were included in the model for older women. For every unit increase in the relative expression of *STAT2* and *RNF4* genes, the odds of having lung cancer dropped by 74% and 84%, respectively. In contrast, the higher the relative expression of *MCM4* gene in older women, the more likely they were to have lung cancer (OR = 6.31).

Logistic models for the male subpopulations showed that the expression of *CPEB4*, *EIF2S3*, and *GRB2* genes was additional age-dependent. *EIF2S3* gene expression was a significant protector in the model for younger men, with the odds of having lung cancer decreasing by 79%. Finally, higher relative expression levels of the *CPEB4* gene (OR = 5.00; StdEst = 0.97) and *GRB2* gene (OR = 20.33; StdEst = 1.37) presented significant risk for the development of NSCLC in older men.

### Cross-validation for logistic models using age/gender-stratified samples

Leave-one-out validation resulted in the selection of identical gene signature for each stratified sample. For older subjects, both men and women, all selected markers were 100% significant in all leave-one-out training models. For younger subjects, both men and women, three markers showed slight difference in significance in leave-one-out training models in comparison with original stratification models: (a) For younger women, *STAT2* was selected as significant for 90% of training models. (b) For younger men, *POLDIP2* and *EIF2S3* were significant for 98.1% and 81.5% of training models, respectively.

The average AUC for leave-one-out training models for each stratified sample (younger women, older women, younger men, and older men) was excellent (0.907, 0.930, 0.896, and 0.970, respectively). The corresponding standard errors were very small, ranging from 0.003 to 0.019. For each age/gender-stratified sample, three or four cutoff values were chosen to evaluate the predicative accuracy of leave-one-out training models (Supplementary Table S2). We took the cutoff value 0.5 to illustrate our findings. For women and younger men, the average sensitivity was higher than 76% and higher than 82%, respectively, while the average specificity was 90% and 83%, respectively. The average accuracy was 85% for women and younger men. Finally, all three indices were higher than 85% for the older men.

### Stage-dependent NSCLC-associated molecular markers

Two case–control subpopulations, including early-stage cases (stage I–IIIA) vs. all non-cancer controls and advanced stages (stage IIIB–IV) vs. all non-cancer controls, were investigated. The logistic models resulted in the selection of six and eight significant NSCLC-associated markers in the early-stage (Table 5) and advanced-stage (Table 6) subpopulations, respectively. Particularly, six genes identified in the early-stage model were also included in the advanced-stage model and the LCM model (Table 3). Although the trend for the classification was similar for the two stage-dependent models, the magnitudes of the expression of *DUSP6*, *GRB2*, and *MDM2* genes were very different on the basis of their odds ratios. For every unit increase in the relative expression of *DUSP6* and *GRB2* genes, the odds of early-stage disease increased by 5.43 and 5.52, whereas the odds of advanced-stage disease increased by 12.49 and 20.48, respectively. Moreover, for every unit increase in the relative expression of *MDM2* gene, the odds of having early-stage lung cancer increased by 12.36 and 4.43 for advanced-stage disease. In addition, the expression of *MDM2* gene had the strongest effect for the early-stage model (StdEst = 0.76, *p* < 0.0001), whereas the expression of *GRB2* gene was the most significant factor for the advanced-stage model (StdEst = 1.29, *p* < 0.0001).

**Table 5:** Multivariate analysis of NSCLC-associated molecular markers based on stage-stratified subpopulations containing early-stage (I–IIIA) NSCLC cases (n = 59) and all non-cancer controls (n = 310). #*

Variable	OR	95%CI of OR		*p*	StdEst
*DUSP6*	5.43	2.46	12.01	0.0000	0.67
*GRB2*	5.52	2.28	13.36	0.0002	0.70
*MDM2*	12.36	4.01	38.09	0.0000	0.76
*EIF2S3*	0.19	0.07	0.57	0.0028	−0.42
*POLDIP2*	0.16	0.06	0.44	0.0004	−0.68
*RNF4*	0.21	0.06	0.74	0.0149	−0.49
C statistic	0.883				

OR: odds ratio; CI: confidence interval; StdEst: standardized coefficients;

# The multiple logistic model contains 15 expressed genes, with controlling for age, gender, and smoking status. The other seven molecular markers (*CPEB4*, *EXT2*, *IRF4*, *MCM4*, *MMD*, *NF1*, *STAT2*, *WEE1*, and *ZNF264*) were not significantly associated.

* The performance of this model is presented as the sensitivity and specificity depending on the cut-off value chosen:

cut-off value =0.391, Sensitivity = 0.661, Specificity = 0.952

cut-off value =0.224, Sensitivity = 0.763, Specificity = 0.900

cut-off value =0.172, Sensitivity = 0.763, Specificity = 0.855

cut-off value =0.127, Sensitivity = 0.797, Specificity = 0.800

cut-off value =0.100, Sensitivity = 0.847, Specificity = 0.735

**Table 6:** Multivariate analysis of NSCLC-associated molecular markers based on stage-stratified subpopulations containing advanced-stage (IIIB-IV) NSCLC cases (n = 128) and all non-cancer controls (n = 310). #*

Variable	OR	95%CI of OR		*p*	StdEst
*DUSP6*	12.49	5.55	28.12	0.0000	1.10
*GRB2*	20.48	7.12	58.91	0.0000	1.29
*MDM2*	4.43	1.61	12.16	0.0039	0.52
*EIF2S3*	0.23	0.08	0.67	0.0066	−0.42
*POLDIP2*	0.10	0.04	0.30	0.0000	−0.87
*RNF4*	0.25	0.08	0.82	0.0228	−0.45
*STAT2*	0.32	0.12	0.85	0.0219	−0.35
*WEE1*	0.29	0.13	0.62	0.0017	−0.46
C statistic	0.953				

OR: odds ratio; CI: confidence interval; StdEst: standardized coefficients;

# The multiple logistic model contains all 15 expressed genes, with controlling for age, gender, and smoking status. The other seven molecular markers (*CPEB4*, *EXT2*, *IRF4*, *MCM4*, *MMD*, *NF1*, and *ZNF264*) were not significantly associated.

* The performance of this model is presented as the sensitivity and specificity depending on the cut-off value chosen:

cut-off value =0.514, Sensitivity = 0.781, Specificity = 0.952

cut-off value =0.311, Sensitivity = 0.859, Specificity = 0.903

cut-off value =0.187, Sensitivity = 0.914, Specificity = 0.852

cut-off value =0.125, Sensitivity = 0.953, Specificity = 0.768

Moreover, the *STAT2* (OR = 0.32) and *WEE1* genes (OR = 0.29) were identified as NSCLC-significant protective markers for the advanced-stage model, but not for the early-stage model. Finally we noticed that the expression of *STAT2* gene became significant in the advanced-stage model rather than the transcript of the *NF1* gene for the LCM model.

Detection of the risk of developing lung cancer by stage-dependent models (early stage and advanced stage) achieved the performance with AUC of 0.883 and 0.953, respectively (Tables 5 and 6).

## DISCUSSION

Early detection improves the survival of patients with lung cancer. Several biomarkers have been identified by analyzing tumor specimens obtained by biopsy or surgical resection during initial diagnosis [12]; however, in clinical practice, blood samples are more feasible for biomarker evaluation. Assessment of gene expression from whole blood or PBMCs has been documented as a valuable method for lung cancer diagnosis and prognosis [20–24]. Several mRNA markers in the peripheral blood of patients with NSCLC were tested by PCR with limited sensitivity [13–18, 25]. Those that were more representative NSCLC classifiers, such as the PBMC-based 29-gene panel and the whole blood-based 484 NSCLC-specific features, were able to distinguish patients with NSCLC from individuals with non-malignant lung disease [20, 23].

We identified 11 quantitative PCR-based NSCLC-associated markers using logistic regression analyses in our age- and gender-matched case–control study; these included *CPEB4*, *DUSP6*, *EIF2S3*, *GRB2*, *MCM4*, *MDM2*, *NF1*, *POLDIP2*, *RNF4*, *STAT2*, and *WEE1*. These statistically significant markers in our study were very different from those of other blood-based studies. One major reason may be the use of lung tumor tissue as the initial screening for candidate genes. In general, in tumor tissue, cancer cells are more abundant than immune cells; thus, those preselected candidate genes were more likely to be direct cancer-associated markers in our PBMC study. In contrast, immune cells are the dominant cell type for gene expression profiling when the whole blood or PBMC fractions are used as the initial screening material. Cancer-related immune responses are expected to be the predominantly detected signals. Previously identified NSCLC classifiers using PBMC and whole blood as initial screening materials were enriched in immune-associated genes [20, 23]. The authors assumed that cancer cells secreted cytokines and/or immune factors and that the communication between immune and cancer cells led to the altered gene signatures in patients with NSCLC compared with the normal subjects.

In contrast to findings of other blood-based studies, we found at least four NSCLC-associated markers that were involved in Ras/MAP kinase and cell growth control pathways highly relevant to cancer: *DUSP6*, *GRB2*, *MDM2*, and *NF1* [26–30]. Of the rest, *CPEB4*, *MCM4*, *RNF4*, *STAT2*, and *WEE1* were also shown to correlate with a number of cancer types [12, 31–41]. In addition, five of the genes that we identified in this study, *CPEB4*, *DUSP6*, *NF1*, *RNF4*, and *STAT2*, were previously proposed as prognostic markers for NSCLC [12, 39], whereas changes in the expression of *MCM4* and *WEE1* were associated with lung cancer development [33, 41]. Notably, while several aspects in the study design, including the preliminary screening of candidate genes from PBMCs and tissues and the composition of subjects with regard to ethnicity, gender ratios, age, cancer subtype, and stage were different between those studies and ours, one common factor was the utilization of patient PBMCs.

The probability of developing invasive cancer increases with age, from 0.2% to 6.7% from birth to >70 years, respectively, in men and from 0.2% to 4.9%, respectively, in women [42]. However, the molecular basis of age and gender effects on cancer incidence remains unclear. Our age- and gender-stratified study provided the very first information about age- and/or gender-dependent NSCLC-associated markers. First, an increased relative expression of *DUSP6* was a significant NSCLC risk factor in all four subpopulation models, suggesting an essential role for *DUSP6* expression during tumorigenesis and metastasis of NSCLC. Our findings are in agreement with those reported by Lee and colleagues who demonstrated a positive correlation between high *DUSP6* expression and lung adenocarcinoma [43], because the majority of our case subjects (73.8%) had lung adenocarcinoma.

Second, most of the genes investigated in this study exhibited additional correlations with gender and age, such as male dependency (*POLDIP2*), age dependency (*MDM2*, *NF1*, and *WEE1*), and age and gender dependency (*CPEB4*, *EIF2S3*, *GRB2*, *MCM4*, *RNF4*, and *STAT2*). Our analysis results confirmed our presumption that some disease-related factors only for a particular age and/or gender subpopulation. For example, *CPEB4*, *MCM4*, and *STAT2* was specifically selected for older men, for older women, and for younger women, respectively. To the best of our knowledge, there are a limited number of reports on the direct correlations between these molecular markers and demographic factors (i.e., age and gender) of NSCLC patients. For instance, a higher expression of *MDM2* was observed in younger women with breast cancer [44], whereas Adami and colleagues reported positive effects of female sex hormones on the incidence of lung cancer in women receiving hormone replacement therapy, particularly in those with a history of smoking [45]. In addition, Magnussen and colleagues [46] showed a correlation between high *WEE1* expression and vulvar squamous cell carcinoma prevalence in younger patients. One possible reason for the important protective effect of *WEE1* expression in younger subjects is its role in mitotic control in active juvenile cells. Further investigation is warranted to ascertain the correlations between these markers and age and gender.

The severity of primary lung cancer is currently determined by the TNM staging system; however, NSCLC recurrence in certain early-stage patients is common, albeit unpredictable, following surgical tumor resection [47]. Uramoto and Tanaka [48] proposed several factors to explain this phenomenon, including occult micrometastatic cancer cells and circulating tumor cells. We explored PBMC-based stage-dependent gene signatures using stage-stratified subpopulations and specifically identified two advanced stage-dependent genes (*STAT2* and *WEE1*), in addition to six common NSCLC-associated genes (*EIF2S3*, *DUSP6*, *GRB2*, *MDM2*, *POLDIP2*, and *RNF4*). It is intriguing that the expression of *STAT2* was associated specifically with younger female subjects, whereas *WEE1* expression was associated with younger age in both genders in age/gender-stratified analyses. We propose that the expression of *STAT2*, as well as *WEE1*, is more robust as prognostic factors for earlier recurrence and/or distant metastasis in younger NSCLC patients than in older NSCLC; this hypothesis remains to be examined in future work.

The logistic regression models established in our study were useful in the classification of subjects with lung cancer risk. The majority of classification models delivered very good to excellent performance with the area under the receiver operating characteristic curve (AUC) ranging from 0.883 to 0.970. The model for the older male subpopulation was the strongest, which may be due to the larger sample size after stringent controlling for age and gender. Moreover, the cross-validation of logistic models for total sample and for age- and gender-stratified sample confirmed the significance of the selected NSCLC-associated markers and their high predictive accuracy. In addition, the sensitivity for an independent validation sample was 75.86% using the classification model for total sample with the cutoff value at 0.434. More clinical samples should be included for validation as future work. Overall, four characteristics were the most likely contributors to the excellent performance of our classification model. First, the candidate genes were obtained from tumor tissues by initial screening. The second character was the comparable cancer incidence rate between the included control subjects and general population. Finally, matched age and gender between cases and controls, as well as the smoking status were controlled for the logistic model.

There were some limitations in this study. First, the frequency of non-smoker subjects was higher in the non-cancer control group than that in the NSCLC case group, and the study groups were not matched for smoking status. In addition, the smoking amount was not considered in the analysis, and its influence on gene expression in healthy subjects and patients with lung cancer remains unknown. Second, all patients participating in this study were Asian, and utility of the eight-gene classification model, as well as age-, gender-, and stage-dependent signatures, in detecting potential lung cancer cases in other ethnicities requires further investigation. Third, the PBMC-based method may result in lower sensitivity for detection of NSCLC at early stage, especially stage I-II, because the tumor is majorly localized in the lung at this stage.

In conclusion, we demonstrated a promising PBMC-derived method for NSCLC detection based on gene signatures. Our eight-gene classification model may provide a feasible, minimally invasive method for the identification of those patients at a higher risk for developing lung cancer.

## MATERIALS AND METHODS

### Patients, controls and blood samples

In total, 187 patients with clinically confirmed NSCLC were enrolled (June 2006–October 2009) in a prospective investigational protocol, which was approved by the Institutional Review Board at Tri-Service General Hospital (Taipei, Taiwan) (Table 1). Patients with NSCLC at different clinical stages were classified according to the TNM system, American Joint Committee on Cancer 7^th^ edition, including 36 (19.25%) stage I, 8 (4.28%) stage II, 15 (8.02%) stage IIIA, 42 (22.46%) stage IIIB, and 86 (45.99%) stage IV. Pathologically, 138 (73.8%) patients had adenocarcinoma, 14 (7.5%) had squamous cell carcinoma, and 35 (18.7%) had other types of NSCLC.

The non-cancer controls were 310 volunteers who had come to our institution for a routine health examination during November 2005–November 2010. They had no evidence of any clinically detectable cancer disease by the time of blood sample collection. The majority of control subjects did not have any suspicious lung nodules by X-ray radiograph during the study period. The follow-up period of 310 controls ranged between 4.8 and 9.9 years. Twenty-six controls (8.39%) were censored, and the status of 284 controls were confirmed as with or without cancer as of September, 2015. Twelve of 284 (4.225%) control subjects were diagnosed with cancer within the follow-up period. More detailed was described in the Supplementary Materials and Methods (Section 1).

An independent validation sample containing 29 cases with early stage (I-II) of NSCLC disease was included. They were enrolled during November 2012 and August 2014 (later than the original study set). The investigation protocol was approved by the Institutional Review Board of the same hospital as for the original sample. Pathologically, there were 23 (79.31%) adenocarcinoma, three squamous carcinoma, one large cell carcinoma, and two NSCLC.

Samples of peripheral blood (6–8 mL) were drawn after obtaining written informed consent from the healthy volunteers and patients with NSCLC before any treatment. Blood samples were stored at 4°C until the peripheral mononuclear cell (PBMC) fraction was isolated, within 3 hours of collection. The PBMC fraction was used for further preparations according to Chang et al [49], including RNA extraction, reverse transcription, and real-time PCR analysis. All RNA and cDNA were stored at −80°C before analysis.

### Cell cultures

The human lung adenocarcinoma cell line A549 was obtained from Bioresource Collection and Research Center (Hsinchu, Taiwan; Cat. No. BCRC60074), and maintained in Ham's F-12K medium with 2 mM glutamine adjusted to contain 1.5 g/L sodium bicarbonate supplemented with 10% fetal bovine serum. The cells were incubated in 5% CO_2_ humidified at 37°C for growth. Cell cultures were split 10-fold every 3–4 days.

### Validation of reference genes for real-time PCR assay

Two commonly used reference genes, *HPRT1* (hypoxanthine phosphoribosylthansferase) and *ACTB* (beta-actin), were evaluated for relative quantitative real-time PCR assay. The human lung adenocarcinoma cell line A549 was spiked into healthy volunteer blood samples at concentrations of 0, 50, 100, 300, 1000 and 3000 cells/mL, and processed to isolate the PBMC fraction. Total RNA extraction, cDNA synthesis, and real-time PCR assay were performed using pre-designed, gene-specific amplification primers, including HK-HPRT1 Primer and HK-ACTB Primer (Advpharma, Taiwan). The means of five repeated measurements (cycle number; Cp values) derived from graded A549-spiked samples were 28.84 ± 0.57 for the *HPRT1* gene and 18.50 ± 0.77 for *ACTB* gene. The Cp values of *HPRT1* (written as Cp[*HPRT1*]) presented with a medium–low expression level, consistent with the report by Dheda et al [50]. The *HPRT1* was used as reference gene for further real-time PCR assays, because the Cp values of the investigated genes in our study had medium–low expression levels.

The expression of each investigated gene in a sample or cell was normalized to that of the *HPRT1* gene and presented as a delta Cp value (Cp[*HPRT1*] − Cp[investigated gene]), which is inversely correlated with the gene expression [51].

### Assessment of the sensitivity of the blood-based molecular assay

We used A549 cell-spiked blood samples to mimic blood samples derived from patients with lung cancer to evaluate the detection sensitivity of the current assay. The A549 cells were added into whole blood, with decreasing doses from 500–20 cells/mL. The blood sample that was not spiked with A549 cells was used as the reference.

All blood samples (with and without spiked A549 cell) were simultaneously processed to isolate the PBMC fraction and for total RNA extraction. The correlation between the A549 cell number in the spiked blood sample and expression of keratin 19 (*KRT19*), an A549-specific molecular marker [14, 18, 52], was determined by real-time PCR using KRT19 Primer (Advpharma, Taiwan). The expression of *HPRT1* gene was used as a reference for normalization. A reaction mixture without cDNA was used as a negative control to confirm PCR assay quality for each analytic batch.

Each reaction mixture contained 1/20 volume of cDNA derived from 500–20 cells/mL blood. Therefore, the cell number equivalent was 25, 15, 5, 2.5, 2, 1.5 and 1 cell/assay. The mean Cp(HPRT1) from all tested samples was 25.28, with a standard deviation of 0.21. This test demonstrates that the *HPRT1* gene is a valid reference gene for our study and provides consistent Cp values irrespective of the cancer cell number in the sample. To assess the detection performance, the Cp(KRT19) of each sample was firstly normalized with the Cp(HPRT1), and the delta Cp (Cp[KRT19] − Cp[HPRT1]) was obtained. Then, the relative change for each spiked sample was calculated as delta − delta Cp = delta Cp(reference) − delta Cp(spiked sample).

In summary, A549 cells at concentrations of over 5 cells/mL blood were detected consistently using the assay method described.

### Preliminary selection of genes for investigation

Nineteen genes were selected at the beginning of this study based on previous studies. Twelve of these genes had a hazard ratio >1, including *CPEB4*, *DLG2*, *DUSP6*, *ERBB3*, *HGF*, *HMMR*, *IRF4*, *MMD*, *RNF4*, *NF1*, *STAT2*, and *ZNF264* [12]. Expression of these genes were considered as risk factors and were associated with a hazard ratio for death from any cause or recurrence of cancer. Another seven expressing genes were identified as significant factors affecting the incidence of NSCLC (unpublished results) including *EIF2S3*, *EXT2*, *GRB2*, *MCM4*, *MDM2*, *POLDIP2*, and *WEE1*.

Real-time PCR assays of 19 genes were performed on a small number (approximately 10) of blood samples for the evaluation of PCR specificity and the reproducibility of measurements using pre-designed, gene-specific primer sets purchased from Advpharma, Taiwan. These primer sets were experimentally validated with the following criteria: (i) a single gene-specific amplified product was confirmed by DNA gel electrophoresis; (ii) the amplification efficiency ranged between 90% and 95%; and (iii) the Cp-value was less than 30. After this preliminary test, the real-time PCR measurements of 15 genes were found to fulfil the criteria and were thus applied for further investigation, including *CPEB4*, *DUSP6*, *EIF2S3*, *EXT2*, *GRB2*, *IRF4*, *MCM4*, *MDM2*, *MMD*, *NF1*, *POLDIP2*, *RNF4*, *STAT2*, *WEE1*, and *ZNF264*.

### Statistical analysis

Chi-square and independent two-sample *t*-tests were first used to evaluate the bivariate associations between the demographics and lung cancer status, as well as between relative gene expression and lung cancer status. We used logistic regression model for estimation of the probability of occurrence of an event, such as lung cancer (Supplementary Materials and Methods; Section 2).

Model fitness was assessed by a receiver operating characteristic (ROC) curve which was created by plotting the true positive rate (TPR; sensitivity) against the false positive rate (FPR; 1-specificity) at various threshold settings. The area under the ROC curve (AUC), which is C statistic, was regarded as an index for the performance of the model. The impact level of the covariates was evaluated by standardized coefficient (StdEst). Models are typically considered to be reasonable when the *C* statistic is higher than 0.7 and strong when *C* exceeds 0.8 [53].

In order to explore age- and/or gender-dependent cancer molecular markers, we stratified participants into two different sexes (men and women), and then further into two age subpopulations with a cut-off age of 65 years. Those older than 65 years old are considered by the World Health Organization as “elderly” or older persons in most developed countries. Moreover, NSCLC cases were stratified into early-stage (clinical stage I-IIIA) and advanced-stage (clinical stage IIIB-IV). These subpopulations were applied for selection of stage-dependent markers.

Classification process can be performed for a given cutoff value. For example, a cutoff value could be set to equal to 0.5. A subject was classified as a case if the probability is greater than 0.5, and was classified as a control if the probability is smaller than 0.5.

Cross-validation was applied to evaluate how well the classification model generalized. (A) Repeated random sub-sampling method was used for total sample: The total dataset (*N* = 497) was randomly split into the training dataset (N = 447; 90%) and testing dataset (*N* = 50; 10%). For each split, the model was fit to the training dataset, and significant factors were selected for each training model. This random sub-sampling and modeling was repeated for 100 times. The predictive accuracy was then assessed using the testing data. The results of predictive accuracies were then averaged over 100 subsamples. (B) Leave-one-out method was applied for age/gender-stratified samples: One dataset from the age/gender-stratified samples was left out for the leave-one-out model validation, and the rest was used as training dataset. Logistic regression analysis was then performed using the training dataset, including selected significant genes and a control variable (smoking status). The procedure was then continued until all samples were selected as a test sample. Finally, the quality and appropriateness of our study design and inclusion of cases and controls were confirmed according to textbooks written by Schulz and Grimes [54] and by Dawson and Trapp [55].

## SUPPLEMENTARY MATERIALS FIGURES AND TABLES


