# Supplementary material for: Panels of tumor-derived RNA markers in peripheral blood of patients with non-small cell lung cancer: their dependence on age, gender and clinical stages
Source: Oncotarget. 2016 Jul 13;7(31):50582–95. doi: 10.18632/oncotarget.10558 (PMC5226605; doi:10.18632/oncotarget.10558)
Supplement: Supplementary file 1 [file oncotarget-07-50582-s001.pdf]

Panels of tumor-derived RNA markers in peripheral blood of patients with non-small cell lung cancer: their dependence on age, gender and clinical stages

SUPPLEMENTARY MATERIALS AND METHODS

Section 1: Follow-up investigation of incidental cancer in control subjects

The follow-up investigation of controls were additionally performed in September 2015. There are 26 controls (8.39%) censored and 284 controls confirmed their status (with or without cancer disease). Twelve controls (4.225%) of 284 diagnosed with cancer: Five controls (1.760%) had cancer disease during the follow up period up to 5 years. Seven controls (2.465%) had cancer disease during the follow up period of 5-9.9 years. The cancer types of these 12 controls included adenocarcinoma lung cancer (1), bladder cancer (1), breast cancer (3), ovarian cancer (1), colon cancer (1), hepatoma (1), urothelial cell carcinoma of renal pelvis (1), B cell lymphoma over bilateral adrenal gland (1), B cell lymphoma of stomach (1) and prostate cancer (1).

Section 2: Logistic regression model

The logistic regression model gives the probability that the event (lung cancer) occurs as an exponential function of independent variables. The model is written in terms of a probability (risk score), which is in the range of 0 to 1, that is

P[The event occurs] = exp(Y)/(1+exp(Y)).

In our study, Y is defined as

$$Y = \beta_0 + \beta_1\text{Gene}_1 + \beta_2\text{Gene}_2 + ... + \beta_k\text{Gene}_k + \beta_{k+1}\text{AGE} + \beta_{k+2}\text{Gender} + \beta_{k+3}\text{SmokingStatus},$$

where β_0 is called the "intercept" and β_1, β_2, β_3 and so on, are called the regression coefficients of AGE, Gender, SmokingStatus, GENE_1, GENE_2,… GENE_k, respectively.

SUPPLEMENTARY FIGURE AND TABLES

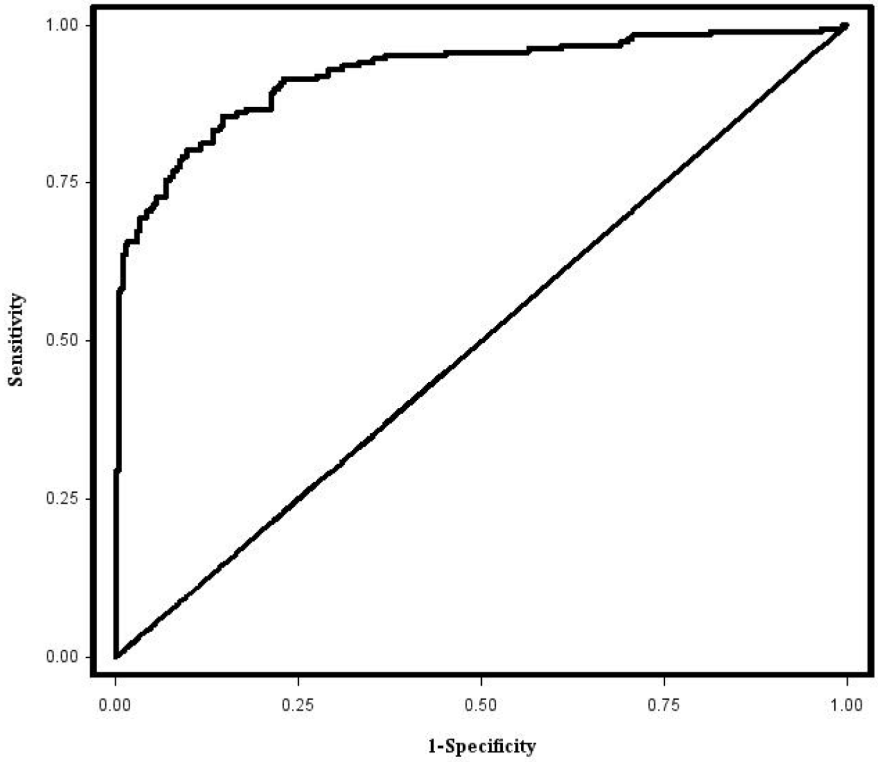

Supplementary Figure S1: Graph of a receiver operating characteristic (ROC) curve. This curve is based on the multiple logistic model (LCM) shown in Table 3, with an area under the curve (AUC) of 0.924.

Supplementary Table S1: Predictive accuracy of training models for repeated random sub-sampling validation

Cutoff	Sensitivity	Specificity	PPV	NPV	Accuracy
0.622	73.1%	90.8%	82.97%	84.6%	84.1%
0.500	76.1%	87.7%	79.15%	85.7%	83.3%
0.434	77.5%	86.0%	77.28%	86.2%	82.8%
0.321	81.7%	82.1%	73.26%	88.0%	81.9%
0.226	86.5%	76.6%	69.38%	90.2%	80.4%

Five cutoff values were chosen to evaluate the predictive performance of training models, including sensitivity, specificity, positive predictive value (PPV), negative predictive value (NPV), accuracy (percent of corrects).

Supplementary Table S2: Averaged performance of leave-one-out cross-validation of age/gender-stratified training models

(A) Younger women	Sensitivity		Specificity		Accuracy	
Cutoff value	Mean	STD	Mean	STD	Mean	STD
0.208	0.924	0.007	0.657	0.010	0.761	0.007
0.497	0.762	0.019	0.901	0.004	0.847	0.019
0.637	0.588	0.024	0.937	0.007	0.801	0.024

(B) Older women	Sensitivity		Specificity		Accuracy	
Cutoff value	Mean	STD	Mean	STD	Mean	STD
0.358	0.822	0.009	0.875	0.008	0.858	0.009
0.401	0.794	0.010	0.891	0.006	0.860	0.010
0.448	0.766	0.010	0.917	0.004	0.869	0.010
0.482	0.765	0.008	0.918	0.004	0.869	0.008

(C) Younger men	Sensitivity		Specificity		Accuracy	
Cutoff value	Mean	STD	Mean	STD	Mean	STD
0.241	0.952	0.003	0.683	0.008	0.788	0.003
0.341	0.860	0.012	0.774	0.011	0.807	0.012
0.421	0.806	0.011	0.833	0.007	0.822	0.011
0.549	0.715	0.015	0.887	0.010	0.820	0.015

(D) Older men	Sensitivity		Specificity		Accuracy	
Cutoff value	Mean	STD	Mean	STD	Mean	STD
0.378	0.944	0.003	0.908	0.003	0.922	0.003
0.414	0.918	0.006	0.925	0.006	0.922	0.006
0.484	0.866	0.011	0.938	0.005	0.909	0.011
0.500	0.862	0.006	0.946	0.005	0.913	0.006

For each age/gender-stratified sample, three to four cutoff values were chosen to evaluate the predicative accuracy of leave-one-out training models. Three indices including the average sensitivity, the average specificity and the average of accuracy (percent of corrects), as well as the corresponding standard errors (STD), were computed. Cross-validation results are listed for (A) Young women subpopulation; (B) Older women subpopulation; (C) Younger men subpopulation and (D) Older men subpopulation.
